# Coevolution of NK and Tumor Cell States Along Multiple Myeloma Progression from Precursor Conditions

**DOI:** 10.3390/ijms27114682

**Published:** 2026-05-22

**Authors:** Cristina Aquilina, Andrea Romano, Anna Maria Corsale, Marta Biondo, Maria Speciale, Elena Tofacchi, Marta Di Simone, Emilia Gigliotta, Costanza Dieli, Claudia Avellone, Angelo Toscano, Lawrence Camarda, Alessandra Romano, Daniela Cambria, Gianluca Giavaresi, Lavinia Raimondi, Antonino Neri, Stefania Campana, Nadia Caccamo, Francesco Dieli, Sergio Siragusa, Serena Meraviglia, Cirino Botta

**Affiliations:** 1 Department of Health Promotion, Mother and Child Care, Internal Medicine and Medical Specialties (PROMISE), University of Palermo, 90127 Palermo, Italy; cristina.aquilina@unipa.it (C.A.); annamaria.corsale@unipa.it (A.M.C.); elena.tofacchi@unipa.it (E.T.);; 2 Central Laboratory of Advanced Diagnosis and Biomedical Research (CLADIBIOR), University of Palermo, 90127 Palermo, Italy; andrea.romano02@unipa.it (A.R.); costanza.dieli@unipa.it (C.D.);; 3 Department of Surgical, Oncological and Stomatological Disciplines (DICHIRONS), University of Palermo, 90127 Palermo, Italy; 4 Hematology Section, Department of General Surgery and Medical-Surgical Specialties, University of Catania, 95123 Catania, Italy; 5 General Orthopedics, IRCCS Rizzoli Orthopedic Institute, 40136 Bologna, Italy; 6 Department of Orthopaedics and Traumatology, University of Palermo, 90127 Palermo, Italy; 7 Surgical Sciences and Technologies, IRCCS Rizzoli Orthopedic Institute, 40136 Bologna, Italy; gianluca.giavaresi@ior.it (G.G.); lavinia.raimondi@ior.it (L.R.); 8 Scientific Directorate, Azienda USL-IRCCS of Reggio Emilia, 42122 Reggio Emilia, Italy; 9 Department of Clinical and Experimental Medicine, University of Messina, 98125 Messina, Italy; 10 Department of Biomedicine, Neuroscience and Advanced Diagnosis (Bi.N.D.), University of Palermo, 90127 Palermo, Italy

**Keywords:** natural killer cells, multiple myeloma, single-cell RNA sequencing, NK CD56^bright^

## Abstract

Multiple myeloma (MM) develops through asymptomatic precursor stages characterized by progressive remodeling of the bone marrow (BM) immune microenvironment and disruption of bone homeostasis. To delineate changes in natural killer (NK) cell states during disease evolution, we investigated coordinated immune-tumor remodeling by integrating NK cell functional states with plasma cell-intrinsic susceptibility programs derived from CRISPR-based screens across healthy donors (HD), monoclonal gammopathy of undetermined significance (MGUS), smoldering MM (SMM), and newly diagnosed MM patients. The integration of NK cell state-associated gene signatures with plasma cell transcriptional programs revealed stage-specific co-variation between immune and tumor compartments. Public single-cell RNA sequencing datasets were interrogated to resolve NK cell heterogeneity, identifying cytotoxic CD56^dim^ and regulatory CD56^bright^ subsets. NK cell dynamics displayed stage-dependent changes, with early expansion followed by the contraction of CD56^dim^ cells in BM, whereas CD56^bright^ cells showed predominantly compositional remodeling. Within the CD56^bright^ subset, transcriptional changes included an increased expression of *KLRC1* (encoding NKG2A), subsequently validated by multiparametric flow cytometry. In parallel, plasma cell programs associated with NK sensitivity progressively decreased along disease stages, supporting tumor adaptation to immune pressure. The NKG2A ligand HLA-E displayed selective expression within CD16^+^ monocytes and followed a distinct variable pattern across disease stages, highlighting a microenvironmental contribution to NK cell regulation. Collectively, these findings indicate a coordinated process of immune-tumor co-evolution, characterized by dynamic remodeling of NK cell states and plasma cell susceptibility, with the NKG2A–HLA-E axis emerging as a central interface during MM progression.

## 1. Introduction

Multiple myeloma (MM) is a malignancy of bone marrow (BM) plasma cells characterized by the clonal expansion and overproduction of monoclonal immunoglobulins, leading to organ damage and profound immunosuppression [1,2]. Clinically, overt MM is invariably preceded by asymptomatic precursor conditions, including monoclonal gammopathy of undetermined significance (MGUS) and smoldering multiple myeloma (SMM), which represent distinct stages along a continuum of disease evolution [3]. A hallmark of symptomatic MM is myeloma-associated bone disease, driven by a profound disruption of normal bone remodeling characterized by increased osteoclast activity and suppressed osteoblast function. This imbalance results in progressive osteolytic bone destruction, leading to skeletal-related events, including pathological fractures, bone pain, hypercalcemia, and functional impairment [4]. Progression from MGUS to SMM and ultimately to symptomatic MM reflects a complex evolutionary process driven not only by the accumulation of genetic alterations within plasma cells but also by progressive remodeling of the BM microenvironment, disruption of bone homeostasis, and impairment of immune surveillance [5].

Within this context, natural killer (NK) cells play a central role as key effectors of innate immunity, endowed with the ability to recognize and eliminate transformed cells independently of antigen specificity. NK cells are therefore thought to contribute to the early immune control of premalignant and malignant plasma cells. However, accumulating evidence indicates that NK cell function is progressively altered in several tumors [6]. These alterations include the reduced expression of activating receptors such as DNAM-1, NKG2D, and CD16; upregulation of inhibitory checkpoints, including TIGIT, TIM-3, and NKG2A; impaired cytotoxicity; and phenotypic shifts suggestive of dysfunctional or maladaptive states driven by the tumor microenvironment [7]. Despite progressive impairment, NK cells retain significant antitumor potential, as demonstrated by preclinical studies showing that activated or expanded NK cells can efficiently eliminate tumor cells, including clonogenic and drug-resistant populations [8].

Recent studies have further highlighted that these changes are not uniform across the NK compartment, but instead involve subset-specific remodeling and functional diversification of innate lymphocyte states, as observed not only in malignancy but also in other contexts of immune adaptation such as aging and longevity [9]. These observations underscore the importance of context-dependent regulation of immune effector programs, particularly within the BM niche [10]. Among these, CD56^bright^ NK cells have gained increasing attention due to their regulatory properties, cytokine-producing capacity, and heightened sensitivity to inhibitory signaling within the tumor microenvironment [11,12].

A major advance in understanding NK–tumor interactions has recently been provided by single-cell functional genomics approaches. Interestingly, a recent work [13] combined genome-wide CRISPR–Cas9 perturbation screens with single-cell transcriptomic profiling in NK-blood cancer co-culture systems to identify the genetic determinants of tumor cell sensitivity and resistance to NK-mediated killing, demonstrating the central role of NKs in hematological malignancy immune-escape. On these bases, we sought to determine the implication of NK cell remodeling along MM evolution by using single-cell transcriptomics integrated with flow cytometry validation. We define changes in NK cell abundance, subset composition, and functional states driven by the tumor microenvironment to identify potential biomarkers of disease progression and novel therapeutic targets.

## 2. Results

### 2.1. Functional CRISPR Screens Define Plasma Cell Programs Associated with NK Sensitivity and Resistance

To investigate tumor-intrinsic mechanisms regulating susceptibility to NK-mediated cytotoxicity in MM, we derived plasma cell-specific NK sensitivity and resistance signatures from previously published genome-scale LOF and GOF CRISPR–Cas9 screens performed in NK–tumor co-culture systems [13]. Among the seven cell lines included in these studies, we specifically focused on the three MM cell lines (MM1S, KMS11, LP1) to ensure disease relevance. We extracted data related to those specific conditions and integrated them to obtain the two specific sensitivity and resistance signatures, retaining genes consistently identified in at least three out of four experimental settings (including both donor-derived NK cells and the KHYG1 NK cell line for KMS11) (Supplementary Figure S1A,B).

This approach allowed us to retain genes that were consistently identified across multiple experimental conditions.

Among resistance-associated genes, several were directly linked to NK inhibitory regulation and antigen presentation, including *HLA-E* and *TAP1*, consistent with the modulation of inhibitory signaling and MHC class I pathway activity. Additional resistance-related genes included *CFLAR*, associated with the regulation of death receptor-mediated apoptosis, and stress/immune-resistance components such as *PTEN* and *TXNRD1*.

### 2.2. Integrated NK and Plasma Cell Signature Profiling Reveals Condition-Dependent Transcriptional Programs Across Disease Stages

To determine whether previously defined NK-cell functional states could capture biologically meaningful changes associated with disease evolution, we performed an integrated analysis (at patient level) combining NK-cell functional state signatures (resting, cytokine-associated, activated, type I IFN-associated, adaptive) together with tumor-cell sensitivity/resistance programs [13].

This analysis was conducted in a large scRNA-seq BM samples database generated by merging nine publicly available datasets, including 178 patients (54 HD, 25 MGUS, 14 SMM, 85 MM) for a total of 588,747 high-quality cells (see Section 4). Signature scores were computed at single-cell resolution within NK and plasma cells and subsequently aggregated at the patient level, yielding a single value per signature for each patient to enable systematic comparisons across disease stages. This integrated analysis was specifically designed to test whether coordinated variations in NK-cell states and tumor-cell susceptibility could stratify patients along the disease evolution from precursor conditions.

**Figure 1:**
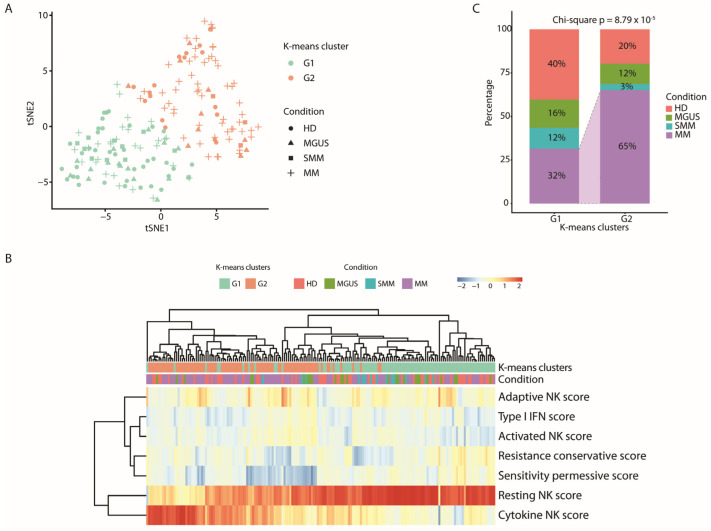
Integrated NK and plasma cell signature profiling across disease stages. (**A**) t-SNE projection of patients based on MM- and NK-associated transcriptional signatures, colored by K-means cluster assignment (G1, G2), with point shapes indicating disease condition. The optimal number of clusters (K = 2) was determined by first selecting a range of potential clusters according to Elbow plot visual inspection of the within-cluster sum of squares across tested K values (range selected: two to eight clusters) and then confirmed by bootstrap-based cluster stability assessment (best option: two clusters), identifying two robust patient groups (G1–G2) from the combined NK cell–plasma cell signature matrix. (**B**) Heatmap showing hierarchical clustering of sample based on the combined enrichment of NK-related functional gene signatures and plasma cell-specific sensitivity and resistance scores across HD, MGUS, SMM, and MM. (**C**) Distribution of clinical conditions across patient groups defined by integrated immune-tumor signature profiling. Stacked bar plot showing the relative proportion of HD, MGUS, SMM, and MM samples within the two patient groups (G1 and G2) identified by unsupervised clustering of combined NK cell and plasma cell transcriptional signatures. Percentages indicate the contribution of each clinical condition within each group.

Unsupervised clustering of patients based on the combined NK cell–plasma cell signature matrix identified two major patient groups (G1–G2) (Figure 1A), indicating that NK cell states and plasma cell sensitivity/resistance programs vary in a coordinated manner during disease progression. Importantly, patient group assignment was significantly associated with clinical conditions (*p* = 8.79 × 10^−5^) (Figure 1C), supporting the biological relevance of this stratification. Group 1 (G1) was characterized by significantly higher enrichment of resting NK signatures together with relatively elevated plasma cell sensitivity scores (Figure 1B). Notably, this cluster predominantly included HD samples (40%) as well as the majority of MGUS/SMM cases (precursor conditions represents indeed the 28% of overall patient population), consistent with a transcriptional landscape associated with preserved immune surveillance and greater tumor immune control.

In contrast, group 2 (G2) displayed a significantly reduced representation of resting NK programs counterbalanced by an increase in cytokine-associated NK signatures and reduced sensitivity-related scores in plasma cells. This group was enriched for MM samples (65%) and a subset of precursor conditions and HD (overall, accounting for 35% of all patients).

To further dissect the tumor-cell component of this coordinated program, we next examined plasma cell sensitivity and resistance scores across disease stages. Both scores were aggregated at the patient level by averaging single-cell values, considering only samples with at least 10 plasma cells, resulting in a final dataset of 43 HD, 18 MGUS, 14 SMM, and 80 MM patients. The sensitivity score significantly decreased from HD to MM (HD vs. MM adjusted *p* = 0.00032), as expected. Surprisingly, the resistance score also showed a progressive decrease across disease stages (adjusted *p* = 0.00083) (Supplementary Figure S1C,D). This seemingly paradoxical reduction of both sensitivity and resistance scores may reflect a broader transcriptional rewiring toward reduced immunogenicity, rather than a linear acquisition of resistance mechanisms.

Together, these findings indicate that disease progression is associated with the coordinated remodeling of NK-cell functional states and tumor-cell susceptibility, supporting a dynamic interplay between NK-cell programs and plasma-cell intrinsic features.

### 2.3. Single-Cell Transcriptomic Profiling of NK Cell Clusters Along MM Evolution

**Figure 2:**
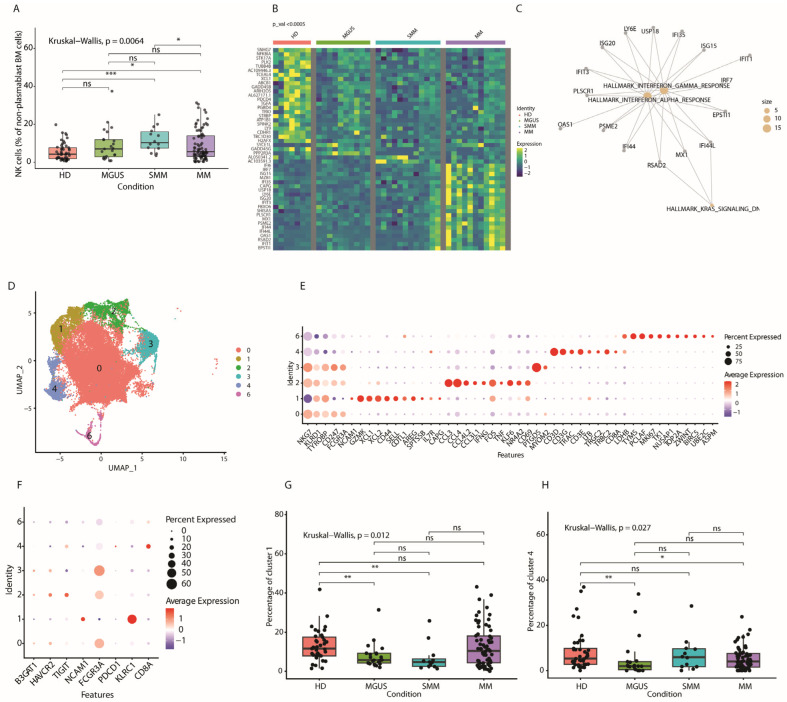
scRNA-seq-based identification, relative abundance of NK cell clusters and pseudobulk gene expression. (**A**) Percentage of NK cells among non-plasmablast BM cells in HD, MGUS, SMM, and MM samples. Each dot represents one sample. Overall differences were assessed by Kruskal–Wallis test (*p* = 0.0064). (**B**) Heatmap of pseudobulk gene expression for significantly differentially expressed genes (*p* < 0.0005) across HD, MGUS, SMM, and MM groups. Columns represent condition-aggregated samples, while rows correspond to the most significant genes. Values are shown as normalized expression levels (color scale from low to high), highlighting distinct expression patterns across disease stages and HD. (**C**) Network representation of enriched MSigDB Hallmark gene sets associated with genes upregulated in MM, highlighting HALLMARK_INTERFERON_ALPHA_RESPONSE, HALLMARK_INTERFERON_GAMMA_RESPONSE, and HALLMARK_KRAS_SIGNALING_DN, together with their contributing interferon-stimulated genes (ISGs). Nodes represent either Hallmark gene sets or individual genes, and node size reflects the number of associated genes. (**D**) UMAP representation of NK cells following RPCA-based integration, showing transcriptionally distinct NK cell clusters identified by unsupervised clustering. (**E**) Dot plot showing typical NK cell genes in addition to the most significant differentially expressed markers identified between clusters. (**F**) Dot plot highlighting the expression of key NK markers associated with functional subsets. (**G**) Relative abundance of Cluster 1 (NK CD56^bright^) cells within the total NK cell compartment across clinical conditions. Mean values (expressed as percentage per patient) were HD (13%), MGUS (8.1%), SMM (6.9%), and MM (12.7%). Statistically significant differences were observed between HD vs. MGUS (adjusted *p* = 0.026) and HD vs. SMM (adjusted *p* = 0.021). (**H**) Relative abundance of Cluster 4 within the total NK cell compartment across clinical conditions. Mean values (expressed as percentage per patient; minimum 30 NK cells per sample) were HD (8.6%), MGUS (5.3%), SMM (7.2%), and MM (5.3%). A statistically significant difference was observed specifically between HD and MGUS (adjusted *p* = 0.029). Pairwise comparisons are indicated as follows: ns: *p* > 0.05; *: *p* ≤ 0.05; **: *p* ≤ 0.01; ***: *p* ≤ 0.001.

Based on previously identified changes in the transcriptional co-modulation of NK and plasma cells during disease progression, suggesting a potential role for NK cells in multiple myeloma pathogenesis, we conducted an in-depth single-cell characterization of the NK cell compartment. Firstly, we evaluated NK cells as a unique population; to this aim, the relative abundance of “virtually” sorted NK cells was assessed in samples with ≥200 total BM cells after plasma cell correction (HD n = 51, MGUS n = 23, SMM n = 14, MM n = 83), and significant quantitative differences were observed across disease stages, with mean NK relative abundance rising from 10.5% in HD to 16.6% in MGUS and 19.9% in SMM (HD vs. SMM adjusted *p* = 0.0035), followed by a non-significant decrease in MM (16.8%) (Figure 2A). As these values are computationally inferred from scRNA-seq data, they should be interpreted as estimated cell proportions rather than directly measured frequencies; importantly, quantification by flow cytometry (Supplementary Figure S5) recapitulated the same overall trend.

In parallel, a pseudobulk differential expression analysis of NK cells revealed disease-stage-dependent transcriptional changes. Specifically, in MM patients, genes associated with interferon (IFN) response were significantly upregulated (*p* < 0.0005), including multiple IFN-stimulated genes such as *IFI6*, *IFI35*, *ISG15*, *IRF7*, *USP18*, and *LY6E* (Supplementary Figure S2A). Accordingly, over-representation analysis (ORA) confirmed the significant enrichment of both “hallmark interferon alpha response” and “hallmark interferon gamma response” signatures in MM, indicating the coordinated activation of IFN-associated transcriptional programs (Figure 2B,C, Supplementary Figure S2B).

Next, we focused on resolving NK cell heterogeneity at single-cell resolution, revealing six transcriptionally distinct NK cell clusters that capture a spectrum of NK cell states rather than fully discrete functional subsets (Figure 2D,E and Supplementary Figure S3). A baseline NK population, Cluster 0 (cytotoxic/resting CD56^dim^ NK) showed a broad expression of core NK markers (*NKG7*, *KLRD1*, *TYROBP*) together with a moderate expression of cytotoxic effector genes (such as *FCGR3A*, *GZMB* and *PRF1)* and an absence of activation genes. Cluster 1 (CD56^bright^NKG2A^+^ NK) was characterized by an increased expression of *KLRC1*, *GZMK*, *XCL1*, *XCL2*, *SELL*, and *CD44*, together with *IL2RB*, *NFKBIA*, and *CD2*, and displayed a low or absent expression of cytotoxicity-associated genes such as *FCGR3A* and *GZMB*. Cluster 2 (activated inflammatory NK) showed an elevated expression of inflammatory/activation genes (*CCL3*, *CCL4*, *CCL4L2*, *CCL3L1*, *IFNG*), early activation genes (*FOS*, *TNF*), and additional activation-associated markers, including *KLF6*, *CD69*, *TNFAIP3*, and *NEAT1*. Cluster 3 (PTGDS^+^ NK) was distinguished by elevated *PTGDS* and *MYOM2* expression, together with increased *CXCR1*, *PRF1*, and *FGFBP2* activation markers. Cluster 4 (NKT-like) exhibited the prominent expression of T cell-associated genes (*CD3D*, *CD3E*, *CD3G*, *TRAC*, and *CD8A*) along with canonical NK markers (*NKG7*, *KLRD1*, *TYROBP*, *CD247*, and *FCGR3A*), supporting a mixed transcriptional profile. This combination of T cell and NK-associated features is consistent with an NKT-like phenotype; however, given the absence of a universally accepted transcriptional signature that unequivocally defines bona fide NKT cells in scRNA-seq data, this annotation should be interpreted with caution. Accordingly, we conservatively refer to this population as “NKT-like”. Finally, Cluster 6 (proliferating NK) displayed strong enrichment of cell cycle-related genes, including *MKI67*, *TOP2A*, *BIRC5*, *UBE2C*, *ASPM*, and *TYMS*, consistent with a proliferative state.

To assess disease-stage-dependent shifts in NK subset composition, relative cluster abundance was compared across HD, MGUS, SMM, and MM, considering only samples containing at least 30 NK cells (HD n = 41, MGUS n = 21, SMM n = 12, MM n = 67) (Supplementary Figure S4). While most clusters showed limited variation across conditions, clusters 1 and 4 displayed statistically significant differences in relative abundance. Notably, C1 (CD56^bright^ NKG2A^+^ NK) exhibited higher relative abundance in HD, a significant progressive decrease in MGUS and SMM, followed by a non-significant increase in MM (Figure 2G). C4 (NKT-like) also differed significantly across conditions (Figure 2H), whereas none of the other clusters showed statistically significant variation. Of interest, we observed a trend to increase in C0 (cytotoxic/resting CD56^dim^ NK), mirroring the observed overall increase in absolute BM NKs.

To further dissect the functional relevance of the CD56^bright^NKG2A^+^ NK subset, pseudobulk differential expression analysis was performed (Supplementary Figure S2C). When comparing MM to earlier disease stages, genes upregulated in Cluster 1 showed significant enrichment for the “hallmark interferon alpha response” and “hallmark interferon gamma response” gene sets, mirroring the interferon-associated transcriptional programs identified in the global NK compartment (Supplementary Figure S2D). These findings indicate that the CD56^bright^NKG2A^+^ NK subset not only undergoes quantitative remodeling along disease evolution, but also acquires a distinct interferon-responsive transcriptional profile in MM. 

Given the disease-associated quantitative and transcriptional remodeling of this CD56^bright^NKG2A^+^ subset across disease stages, we next sought to validate these observations by flow cytometry in an independent cohort (Section 2.4).

### 2.4. Flow-Cytometry-Based NK Cell Profiling Across Disease Stages

**Figure 3:**
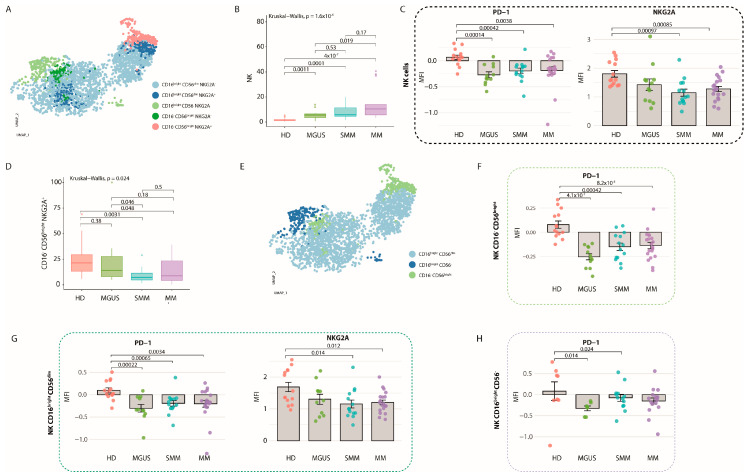
Flow cytometric profiling of bone marrow NK cell subsets across clinical conditions. (**A**) UMAP representation of bone marrow NK cells highlighting cytotoxic and regulatory compartments and resolving NKG2A^+^ and NKG2A^−^ fractions within NK subsets. (**B**) Frequency of total NK cells across conditions. (**C**) PD-1 and NKG2A mean fluorescence intensity (MFI) in total NK cells. (**D**) Frequency of NKG2A^+^ cells within the CD16^−^ CD56^bright^ NK population across HD, MGUS, SMM, and MM. (**E**) UMAP representation of NK cells stratified by CD16^bright^/CD56^bright^ defined subsets. (**F**) PD-1 MFI in CD16^−^ CD56^bright^; (**G**) PD-1 and NKG2A MFI in CD16^bright^ CD56^dim^; (**H**) and PD-1 MFI in CD16^bright^ CD56^−^ NK cells. Data are shown as box plots with individual points representing samples; statistical comparisons are indicated in the figure.

In order to validate transcriptomic findings and to further investigate the distribution of inhibitory receptors (usually difficult to detect at the RNA level), we performed an NK-oriented flow-cytometry screen on 58 patients (12 HD, 12 MGUS, 14 SMM, and 20 MM). First, sub-clustering of gated NK cells revealed a clear separation between cytotoxic and regulatory compartments, with further resolution of NKG2A^+^ and NKG2A^−^ fractions within the main NK populations (Figure 3A). In parallel, stratification based on CD16 and CD56 expression confirmed the segregation of CD16^bright^ CD56^dim^, CD16^−^ CD56^bright^, and CD16^bright^ CD56^−^ NK subsets. Subsequently, we quantified the overall contribution of NK cells to the BM cellular compartment (excluding plasma cells), observing a statistically significant increase in total NK cell frequency from HD to SMM/MM, consistent with our scRNA-seq findings (Figure 3B). Interestingly, an analysis of the mean fluorescence intensity (MFI) of PD-1 on total NK cells showed statistically significant lower values in MGUS, SMM, and MM compared with HD, while MFI of NKG2A showed significant reduction in SMM and MM compared with HD (Figure 3C).

Within the NK compartment, the relative abundance of CD16^−^ CD56^bright^ NKG2A^+^ cells was highest in HD and progressively declined in MGUS and SMM, with a non-significant increase observed in MM, thus validating our previous scRNA-seq findings (Figure 3D, gating strategy reported in Supplementary Figure S5A). Of note, an analysis of PD-1 expression on total NK cells showed significantly lower PD-1 mean fluorescence intensity (MFI) in MGUS compared with HD, while SMM and MM displayed intermediate values (Figure 3F). Subset-specific analyses (Figure 3E) revealed that in the CD16^−^ CD56^bright^ NK cells subset, PD-1 MFI was higher in HD and significantly reduced in the other conditions; meanwhile, in parallel, a similar trend for PD1 was observed in CD16^bright^ CD56^dim^ NK cells (Figure 3F,G) for NKG2A, and MFI showed a decrease from HD to MM (statistically significant for SMM and MM when compared to HD). Finally, in CD16^bright^ CD56^−^ NK cells, a significant reduction for PD1 expression was found for MGUS and SMM patients compared to HD (Figure 3H). No changes were observed in the expression of TIGIT, TIM-3, or CD8 (Supplementary Figure S5B–D). Moreover, given the immunomodulatory properties of CD16^−^ CD56^bright^ NK cells, we investigated their potential association with bone disease (which is a known inflammatory hallmark). A comparison between MM patients with and without bone lesions revealed no significant differences in the distribution of the three major NK cell subsets (CD16^bright^CD56^dim^, CD16^bright^CD56^−^, and CD16^−^ CD56^bright^), as shown in Supplementary Figure S5E. However, stratification according to NKG2A expression revealed a significant increase in the CD16^bright^CD56^dim^NKG2A^−^ subset in patients with bone lesions (*p* = 0.013, Supplementary Figure S5F).

### 2.5. Disease-Stage-Dependent and Coordinated Regulation of BM CD16^+^ Monocyte, HLA-E, and NK Cell NKG2A Expression

Following the identification of disease-associated changes within both the NK and MM cell compartment, highlighting a pivotal role for NKG2A–HLA-E axis modulation along disease evolution, we explored *HLA-E* expression within the different cellular components of the BM microenvironment (Supplementary Figure S6). Of note, HLA-E was one of the highest ranked genes whose knockout in MM cell lines induced an NK-mediated killing in vitro (as previously described, Supplementary Figure S1).

**Figure 4:**
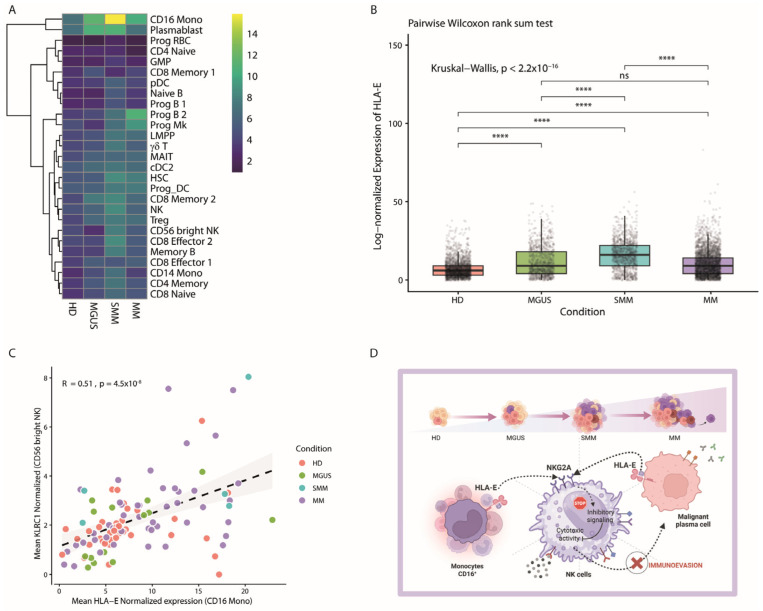
*HLA-E* expression and its relationship with *KLRC1* across disease stages. (**A**) Heatmap of pseudobulk *HLA-E* expression across cell types and conditions. (**B**) Log-normalized *HLA-E* expression levels were assessed in predicted CD16^+^ monocytes from HD (mean = 6.9), MGUS (mean = 11.5), SMM (mean = 15.9), and MM (mean = 10.4). Box plots show the distribution of single-cell expression values, with individual points representing single cells. Overall statistical significance was evaluated using the Kruskal–Wallis test, and pairwise comparisons were performed using Wilcoxon rank-sum tests, as indicated (ns: *p* > 0.05; ****: *p* ≤ 0.0001). (**C**) The scatter plot shows the sample-level correlation between *HLA-E* expression in CD16^+^ monocytes and *KLRC1* expression in CD56^bright^ NK cells, highlighting coordinated modulation of the NKG2A–HLA-E axis across disease stages. (**D**) Schema summarizing the proposed mechanism linking disease progression to NK-cell dysfunction via the HLA-E–NKG2A axis.

Unexpectedly, ranking the different cell types by *HLA-E* expression revealed the highest levels in CD16^+^ monocytes, followed by plasma cells (Figure 4A). Additionally, single-cell expression in CD16^+^ monocytes revealed a condition-dependent distribution of *HLA-E* across disease stages, being significantly lowest in HD, increasing in MGUS, and reaching the highest levels in SMM, followed by a reduction in MM samples, thus indicating a dynamic pattern along disease progression (*p* < 2.2 × 10^−16^) (Figure 4B). To further investigate the relationship between ligand and receptor modulation, a correlation analysis was performed between mean *HLA-E* expression in CD16^+^ monocytes and mean *KLRC1* expression in CD56^bright^ NK cells at the sample level (patients with ≥5 cells of each cell type were retained for a total of 37 HD, 14 MGUS, 5 SMM, and 45 MM). This analysis revealed a significant positive correlation between the two parameters (Pearson’s R = 0.51, *p* = 4.5 × 10^−8^), indicating the coordinated variation of HLA-E expression in monocytes and NKG2A transcriptional levels in CD56^bright^ NK cells across disease conditions (Figure 4C and Supplementary Figure S7), being strongest in the MM setting.

## 3. Discussion

The present work delineates a dynamic and coordinated remodeling of both the BM NK cell compartment and the plasma cell immune-susceptibility landscape throughout MM progression. By integrating CRISPR-derived tumor-intrinsic NK sensitivity/resistance programs, single-cell transcriptomic analyses, and independent flow cytometry validation, we were able to dissect disease evolution as the result of a reciprocal immune-tumor adaptation rather than a unidirectional impairment of NK cells alone. This approach enabled fine discrimination between changes in NK cell subset composition, tissue-level NK abundance, and plasma cell–intrinsic susceptibility to NK-mediated cytotoxicity, identifying the HLA-E–NKG2A axis as a promising biomarker of disease evolution and a potential therapeutic vulnerability.

A key finding of our study is that disease progression is characterized by the coordinated variation of NK functional states and plasma cell sensitivity/resistance programs. Patients enriched for resting NK signatures and higher plasma cell NK-sensitivity scores were predominantly represented by healthy donors and precursor conditions (MGUS/SMM), consistent with preserved immune surveillance. In contrast, overt MM was associated with reduced resting NK programs, increased cytokine- and IFN-associated NK signatures, and lower plasma cell sensitivity scores. These findings indicate that progression from precursor states to symptomatic disease involves both functional rewiring of NK cells and a concomitant shift of plasma cells toward reduced susceptibility to NK-mediated killing, supporting a model of co-evolution between the immune system and tumor cells under selective pressure.

From the immune compartment perspective, both single-cell and flow cytometry analyses consistently demonstrated a disease-stage-dependent expansion of the NK cell compartment within the BM. Early disease stages were characterized by increased NK abundance and the enrichment of cytotoxic transcriptional programs, suggesting an initial phase of active immune surveillance or the onset of inefficient NK-mediated tumor control. However, this expansion did not translate into effective tumor containment and instead culminated in the accumulation of functionally impaired NK cells in advanced disease. The latter is supported by the fact that (1) this expansion was largely driven by the predominant CD56^dim^ NK population, which constitutes the majority of NK cells across all conditions, with the transcriptional shape of resting/partially activated cells; (2) enrichment analyses consistently identified IFN response as dominant signatures in overt disease, indicating widespread activation of IFN-associated transcriptional programs at the BM level; and (3) a coordinated progressive upregulation of both *HLA-E* (on CD16^+^ monocytes and MM cells) and *KLRC1* (on CD56^bright^ NK cells) was observed along disease evolution. Interestingly, rather than reflecting effective immune-mediated tumor control, sustained IFN signaling is consistent with a chronic inflammatory environment that promotes the terminal differentiation and functional impairment of NK cells, thereby contributing to immune dysfunction despite persistent activation cues. Similar patterns of NK cell functional remodeling in the context of chronic immune activation have also been reported in non-malignant inflammatory conditions, where altered NK phenotypes emerge without overt numerical loss [14]. Within this framework, distinct NK subsets exhibited divergent behaviors: while the CD56^dim^ population primarily governed quantitative changes in NK cell abundance, the CD56^bright^ NK compartment underwent pronounced compositional and functional remodeling. Single-cell analyses led us to focus on a CD56^bright^ NK subset characterized by elevated *KLRC1* expression (validated in flow cytometry): although the relative frequency of NKG2A^+^ CD56^bright^ NK cells declined in both MGUS and SMM, *KLRC1* expression within this subset peaked in SMM, indicating the selective reinforcement of inhibitory signaling. Interestingly, the main source of the cognate HLA-E ligand was found to be the CD16^+^ monocytes compartment (rather than MM cells), further supporting the role of the microenvironment in MM progression and evolution [15].

While the association between HLA-E expression and the enrichment of NKG2A-positive NK cells suggests the involvement of this inhibitory axis in MM progression, causality cannot be formally established in this study. However, the convergence of CRISPR-based tumor-intrinsic evidence, single-cell correlation analyses, and independent flow cytometry validation strongly supports the functional role of this axis. Mechanistically, increased HLA-E expression may engage NKG2A on NK cells, thereby suppressing cytotoxic activity and promoting progressive immune escape. Chronic exposure to HLA-E could preferentially restrain NKG2A-expressing NK subsets, ultimately leading to the accumulation of functionally impaired NK cells (Figure 4D). This hypothesis is supported by recent observations in ovarian cancer, where dysfunctional NKG2A^+^ NK cells co-localize with HLA-E^+^ tumor regions, revealing a spatially organized immunosuppressive niche [16]. In addition, the therapeutic blockade of the HLA-E–NKG2A interaction has shown the ability to restore NK cell-mediated cytotoxicity against MM cells in preclinical models [17]. Nevertheless, dedicated functional studies—including ex vivo blockade assays and ligand–receptor interaction analyses—are required to formally validate this mechanism and define its clinical relevance in MM.

On the other hand, we did not observe consistent or biologically meaningful differences in the NK compartment related to major immune checkpoints such as PD1, TIGIT, and LAG3, further supporting the limited efficacy of checkpoint inhibitors in this setting and being in line with reports showing the context-dependent regulation of cytotoxic lymphocytes by immune checkpoint pathways within tumor microenvironments [18]. Although modest variations in PD-1 expression were detected in specific NK subsets, particularly in MGUS, the baseline expression levels remained very low, suggesting that these differences are unlikely to translate into significant biological or therapeutic impact.

In conclusion, our study identifies a progressive shift from an initially activated to a chronically stimulated yet functionally impaired NK cell state during MM evolution, driven by microenvironmental cues rather than tumor-intrinsic mechanisms alone. Importantly, plasma cells concurrently acquire transcriptional features associated with impaired susceptibility to NK-mediated cytotoxicity.

Within this reciprocal adaptation, the HLA-E–NKG2A axis emerges as a central interface linking tumor cells, immune cells, and the BM microenvironment. These findings not only refine the current understanding of NK cell biology in MM but also highlight the HLA-E–NKG2A pathway as a rational and potentially actionable target for next-generation immunotherapeutic strategies aimed at restoring innate immune surveillance by disrupting tumor–microenvironment–NK cell crosstalk.

## 4. Materials and Methods

### 4.1. scRNA-Seq Datasets, Preprocessing, and Cell Type Annotation

Nine publicly available scRNA-seq BM datasets (GSE120221, GSE124310, GSE161722, GSE163278, GSE176131, GSE189460, GSE223060, GSE271107, GSE145977) comprising 178 patients with plasma cell disorders and HD were retrieved from Gene Expression Omnibus (GEO). All patients with MM in these datasets were exclusively newly diagnosed with MM. The composition of the integrated cohort was as follows: GSE120221 (25 HD); GSE124310 (7 HD, 5 MGUS, 10 SMM, 7 MM); GSE145977 (7 HD, 5 MGUS, 10 SMM, 7 MM; plasma cells only); GSE161722 (6 MM); GSE163278 (8 HD, 14 MGUS, 11 MM); GSE176131 (2 HD, 9 MM); GSE189460 (18 MM); GSE223060 (7 HD, 30 MM); and GSE271107 (5 HD, 6 MGUS, 4 SMM, 4 MM). Quality control (QC) was performed in Python (v3.12.4) (Scanpy v1.12). Cells were retained if the mitochondrial gene content was 1–10%, with additional dataset-specific QC to account for sequencing depth and complexity. For each dataset, UMIs and detected genes were modeled on a log–log scale using linear regression (scikit-learn), and outliers from the fitted relationship were removed. Cells with >200 detected genes and <30,000 UMIs were retained. Post-QC data were analyzed in R (v4.5.2) using Seurat v5. Cell type annotation was performed via reference-based mapping using the SeuratData *bmcite* human BM reference. Each dataset was mapped independently (stratified by condition) by projecting query cells into the reference latent space; labels were transferred via nearest-neighbor mapping. For visualization, query cells were projected onto the reference UMAP (no de novo UMAP recomputation), ensuring consistent lineage separation across datasets.

### 4.2. Determination of Plasma Cell NK Sensitivity/Resistance Signatures from Functional CRISPR Screens

Plasma cell-specific gene signatures associated with susceptibility or resistance to NK cell-mediated cytotoxicity were derived from a previously published genome-wide CRISPR–Cas9 screening dataset including both loss-of-function (LOF) and gain-of-function (GOF) perturbations in NK-tumor co-culture systems [13]. To ensure tumor-intrinsic inference, we analyzed four experimental conditions, corresponding to three MM cell lines (namely KMS11, MM1S, and LP1) co-cultured with NK cells from HD-PBMCs, and one MM cell line (KMS11) co-cultured with the KHYG1 NK cell line. For each gene, we assessed whether its perturbation—knockdown in LOF screens or overexpression in GOF screens—consistently increased or decreased NK-mediated killing across conditions, using the directionality defined in the original dataset. Genes whose perturbation increased NK-mediated cytotoxicity were assigned to an NK sensitivity signature, whereas genes whose perturbation reduced NK-mediated cytotoxicity were assigned to an NK resistance signature (Supplementary Figure S1A,B).

To account for inter-line heterogeneity, we applied the following criteria: a concordant effect was required in at least three out of four conditions. The resulting gene sets were subsequently used as plasma cell signatures in downstream single-cell analyses.

Specifically, the NK sensitivity signature comprised *VWF*, *DEFB110*, *IL21R*, *KLK11*, *CTR9*, *SWI5*, *TMEM86B*, *MAPRE3*, *SLC45A2*, *GPR6*, *CCDC92*, *SLC2A4RG*, *ARID5B*, and *USP48*, whereas the NK resistance signature included *PTEN*, *HLA-E*, *TAP1*, *HIRA*, *ARRDC3*, *NT5C1B*, *CFLAR*, *SNTG2*, *NCOA6*, *PCGF6*, *G3BP1*, *PPP1CC*, *RPF2*, *MAN2A1*, *TXNRD1*, *GPR52*, *COX7B*, *SEC61A1*, *DMXL2*, *PTBP1*, *SLC16A1*, *TSC2*, *ZFP36L2*, *RBM22*, *KMT2D*, *PRAC1*, and *LILRB1*.

### 4.3. Patient Stratification Using NK and Plasma Cell Transcriptional Signatures

For population-based analysis at the patient level, “NK cells” population included both “NK” and “CD56 bright NK” clusters after projection on BM reference dataset, while the “plasmablast” population was considered “plasma cells”. NK functional state signatures were derived from Dufva’s work [13] and included resting (*KLRC1, NCAM1, GZMK, GZMA, KLRB1*), adaptive (*KLRC2, GZMH, LAG3, HLA-DRA*), type I IFN response (*ISG15, MX1/2, OAS1–3, IRF7/9, EIF2AK2, LAG3*), activated (*TNFRSF18/9/4, CRTAM, ENTPD1, HAVCR2, TIGIT, TNFSF10, BCL2L11*), and cytokine-producing (*CCL3/4, TNF, IFNG, CD69*). Signature scores were computed at the single-cell level using AddModuleScore function (Seurat R package, v5.4.0) for the five NK programs and the two plasma cell-associated signatures. For each patient, a score for each signature was derived and concurred to the generation of a combined NK cell–plasma cell enrichment score table matrix.

K-means clustering (implemented via the base R function kmeans) was applied to define transcriptional patient groups. To determine the optimal number of clusters, a range of K values (K = 1–50) was explored using the within-cluster sum of squares, identifying a set of plausible solutions on an ElbowPlot (v4.5.2). This was further refined through cluster stability analysis based on bootstrap resampling. Associations between patient groups and clinical conditions were assessed using a chi-squared test.

To evaluate variation in plasma cell susceptibility to NK cell-mediated activity across disease stages, sensitivity and resistance scores were aggregated at the patient level by averaging across plasma cells. Only samples with at least 10 plasma cells were included. Differences across conditions were assessed using the Kruskal–Wallis test followed by adjusted pairwise comparisons.

### 4.4. NK Cell Extraction, Integration, and Subcluster Definition

NK cells were isolated based on reference annotation, retaining “NK” and “CD56 bright NK” only (Supplementary Figure S8). To mitigate batch effects, the RNA assay was split by dataset of origin (GSE) and processed separately prior to integration. Normalization and variance stabilization were applied separately to each GSE using the SCTransform (SCT) method implemented in Seurat. Integration was performed with anchor-based RPCA using Seurat’s IntegrateLayers (3000 HVGs; k.weight = 30). The integrated RPCA reduction was used for downstream analyses (Supplementary Figure S9). Clustering was performed using the first 30 RPCA dimensions (FindNeighbors/FindClusters; Louvain; resolution = 0.1), yielding 10 initial clusters (0–9) visualized by UMAP. Cluster markers were identified with FindAllMarkers (Wilcoxon; min.pct = 0.15; logfc.threshold = 1; adjusted *p* < 0.05) and were used to detect contaminants. Four non-NK clusters were removed: erythroid (*HBA1/2*), myeloid (*CD14*, *MNDA*), B cell (*MS4A1*, *CD79A*), and fibroblast-like (*DCN*, *COL3A1*) (Supplementary Figure S10). Markers for bona fide NK clusters were then refined with FindMarkers (positive markers; adjusted *p* < 0.05) and cluster-specific filtering by avg log2FC and pct.1. Marker visualization was performed by DotPlot, supporting the transcriptional interpretation of NK subpopulations. NK abundance was evaluated per patient as the proportion of NK cells within BMMCs, excluding plasma cells. Analyses were restricted to samples with ≥200 total BM cells after plasma cell correction. Differences across conditions were tested using Kruskal–Wallis with Bonferroni-corrected pairwise Wilcoxon tests. NK subcluster composition was assessed in patients with ≥30 NK cells. For each patient, subcluster abundance was computed as a percentage within total NK cells and compared across conditions using Kruskal–Wallis with multiple-testing corrected post hoc tests.

### 4.5. Pseudobulk Differential Expression, Pathway Enrichment, and Gene Correlations

Pseudobulk differential expression (DGE) was performed on datasets containing all four conditions; thus, GSE124310 and GSE271107 were retained. Raw counts were aggregated at the patient-condition level using Seurat’s AggregateExpression (v5.4.0), generating one profile per patient per condition. For total NK pseudobulk, patients with >20 NK cells were included; for subcluster pseudobulk, analyses were performed per cluster, including patients with ≥4 cells in that cluster. Genes expressed in <10 cells were excluded prior to aggregation. Differential expression across conditions was assessed with DESeq2, considering positive log-fold changes. Given limited power in low-abundance subclusters, nominal thresholds were applied (total NK: *p* < 0.0005; subclusters: *p* < 0.005) for descriptive/hypothesis-generating purposes. For functional interpretation, genes upregulated in MM at both the total NK level and within cluster 1 were tested for MSigDB Hallmark (H) enrichment analysis using clusterProfiler::enricher (v4.18.4; FDR-adjusted *p* < 0.05; universe = all detected pseudobulk genes), with gene sets retrived via msigdbr (v25.1.1). Spearman correlations were computed globally and within each condition; the results were visualized via faceted scatter plots with linear fit overlays.

### 4.6. Patient Enrollment and Flow Cytometry Analysis

The study cohort included HD, MGUS, SMM, and MM patients from the Paolo Giaccone University Hospital in Palermo and the Rizzoli Orthopedic Institute (IOR) in Bagheria. The study was approved by the local Ethics Committee (approval numbers 05/2021 and 02/2022, study codename: MMVision; 02/2023: PNRR-2022 12376660), and written informed consent was obtained from all participants in accordance with institutional guidelines and the Declaration of Helsinki.

BM samples were collected at baseline in EDTA-coated tubes, and the bone marrow mononuclear cells (BMMCs) were isolated by density gradient centrifugation using Ficoll-Paque PLUS (Cytiva, Uppsala, Sweden; cat. #17144003). Isolated cells were subsequently immunophenotypically characterized by multiparameter flow cytometry using a 10-color antibody panel. The panel included antibodies against CD57 FITC (clone NK-1), TIM3 (CD336) PE (clone 7D3), TIGIT BB700 (clone 741182), CD56 PE-Cy7 (clone NCAM 16.2), CD16 APC (clone 3G8), CD279 (PD-1) APC-R718 (clone EH12.1), CD3 APC-H7 (clone SK7), NKG2A (CD159a) BV421 (clone 131411), CD45 V500 (clone HI30), and CD8 BV605 (clone SK1) (all from BD Biosciences, Franklin Lakes, NJ, USA).

Cells were incubated with the antibody cocktail for 20 min at room temperature in the dark, washed, and resuspended in 400 μL of Stain Buffer (BD Biosciences, cat. #554657). Samples were acquired immediately using a BD FACSLyric™ flow cytometer (BD Biosciences), and data were analyzed according to standard gating strategies.

### 4.7. Dimensionality Reduction, Clustering, and Visualization

FlowCT (v1.0.0), a semi-automated workflow for the deconvolution of immunophenotypic data and objective reporting on large datasets, was used for data analysis. The pipeline encompasses data preprocessing, normalization, automated clustering using FlowSOM (v2.10.0) and Seurat (v5.4.0), dimensionality reduction, and predictive modeling integrated with statistical tools. In addition, the framework supports the integration of single-cell analysis tools originally developed for other omics disciplines. For cluster characterization, median marker expression was analyzed on multi-uniform manifold approximation and projection (UMAP) plots, and further validation was performed using Infinicyt software (v2.0.6, Cytognos SL, Salamanca, Spain) to annotate cell populations [19].

## Data Availability

The original contributions presented in this study are included in the article/Supplementary Materials. Further inquiries can be directed to the corresponding authors.

## Supplementary Materials

The following supporting information can be downloaded at: https://www.mdpi.com/article/10.3390/ijms27114682/s1.
