# Supplementary material for: Coevolution of NK and Tumor Cell States Along Multiple Myeloma Progression from Precursor Conditions
Source: Int J Mol Sci. 2026 May 22;27(11):4682. doi: 10.3390/ijms27114682 (PMC13256211; doi:10.3390/ijms27114682)
Supplement: Supplementary file 1 [file ijms-27-04682-s001.zip › ijms-4202461-supplementary.pdf]

Coevolution of NK and Tumor Cell States Along Multiple Myeloma Progression from Precursor Conditions

Cristina Aquilina [1,2,†], Andrea Romano [2,3,†], Anna Maria Corsale [1,2], Marta Biondo [4], Maria Speciale [2,3], Elena Tofacchi [1,2], Marta Di Simone [1,2], Emilia Gigliotta [1], Costanza Dieli [2,3], Claudia Avellone [1,2], Angelo Toscano [5], Lawrence Camarda [6], Alessandra Romano [4], Daniela Cambria [4], Gianluca Giavaresi [7], Lavinia Raimondi [7], Antonino Neri [8], Stefania Campana [9], Nadia Caccamo [2,10], Francesco Dieli [2,10], Sergio Siragusa [1], Serena Meraviglia [2,10,*] and Cirino Botta [1,*]

[1] Department of Health Promotion, Mother and Child Care, Internal Medicine and Medical Specialties (PROMISE), University of Palermo, 90127 Palermo, Italy; cristina.aquilina@unipa.it (C.A.); annamaria.corsale@unipa.it (A.M.C.); elena.tofacchi@unipa.it (E.T.)
[2] Central Laboratory of Advanced Diagnosis and Biomedical Research (CLADIBIOR), University of Palermo, 90127 Palermo, Italy; andrea.romano02@unipa.it (A.R.); costanza.dieli@unipa.it (C.D.)
[3] Department of Surgical, Oncological and Stomatological Disciplines (DICHIRONS), University of Palermo, 90127 Palermo, Italy
[4] Hematology Section, Department of General Surgery and Medical-Surgical Specialties, University of Catania, 95123 Catania, Italy; marta.biondo@unict.it (M.B.)
[5] General Orthopedics, IRCCS Rizzoli Orthopedic Institute, 40136 Bologna, Italy
[6] Department of Orthopaedics and Traumatology, University of Palermo,90127 Palermo, Italy
[7] Surgical Sciences and Technologies, IRCCS Rizzoli Orthopedic Institute, 40136 Bologna, Italy; gianluca.giavaresi@ior.it (G.G.); lavinia.raimondi@ior.it (L.R.)
[8] Scientific Directorate, Azienda USL-IRCCS of Reggio Emilia, 42122 Reggio Emilia, Italy
[9] Department of Clinical and Experimental Medicine, University of Messina,98125 Messina, Italy
[10] Department of Biomedicine, Neuroscience and Advanced Diagnosis (Bi.N.D.), University of Palermo, 90127 Palermo, Italy
* Correspondence: serena.meraviglia@unipa.it (S.M.); cirino.botta@unipa.it (C.B.)
† These authors equally contributed to these works and should be considered as co-first authors.

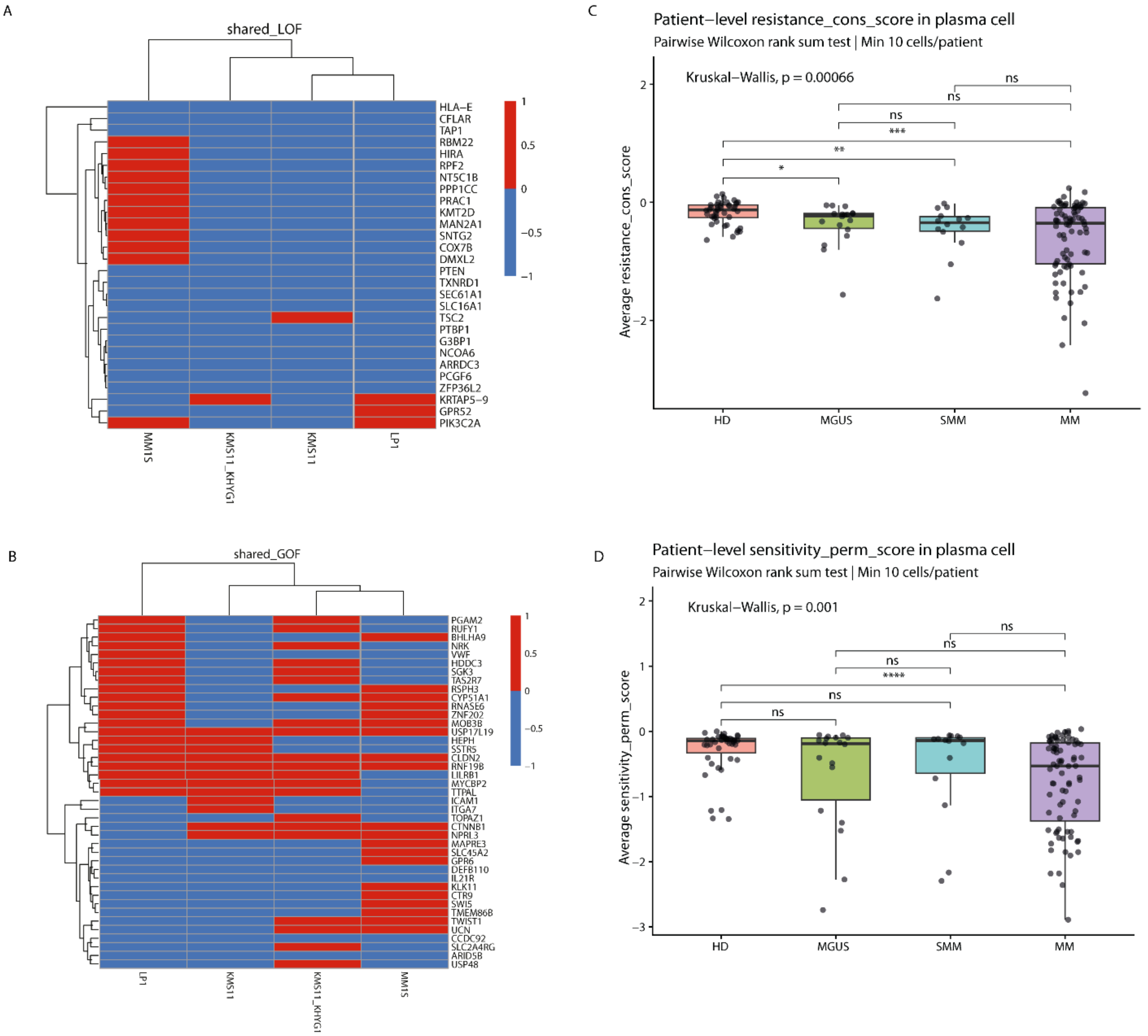

Supplementary Figure S1. ***Shared loss- and gain-of-function transcriptional programs across multiple myeloma cell lines.*** Heatmaps showing genes recurrently associated with **shared loss-of-function (shared_LOF)** (**A**) and **shared gain-of-function (shared_GOF)** (**B**) signatures across MM cell lines (MMIS, KMS11, KMS11_KHYG1, LP1). Gene expression values are shown as scaled z-scores (blue to red indicating low to high relative expression). Hierarchical clustering highlights both conserved and cell line–specific transcriptional patterns, revealing common pathways affected by loss- or gain-of-function events in multiple myeloma. (**C**) Plasma cell resistance score, calculated as the average of single-cell values per patient (≥10 plasma cells), shows a progressive decrease across disease stages (Kruskal–Wallis $p = 0.00066$). Pairwise comparisons are indicated as ns: $p > 0.05$; *: $p \leq 0.05$; **: $p \leq 0.01$; ***: $p \leq 0.001$. (**D**) Plasma cell sensitivity score, computed using the same approach, significantly decreases from HD to MM (Kruskal–Wallis $p \approx 0.001$). Each dot represents an individual patient; boxplots display median and interquartile range. Statistical significance was assessed using Kruskal–Wallis test with post hoc pairwise comparisons that are indicated as ****: $p \leq 0.0001$.

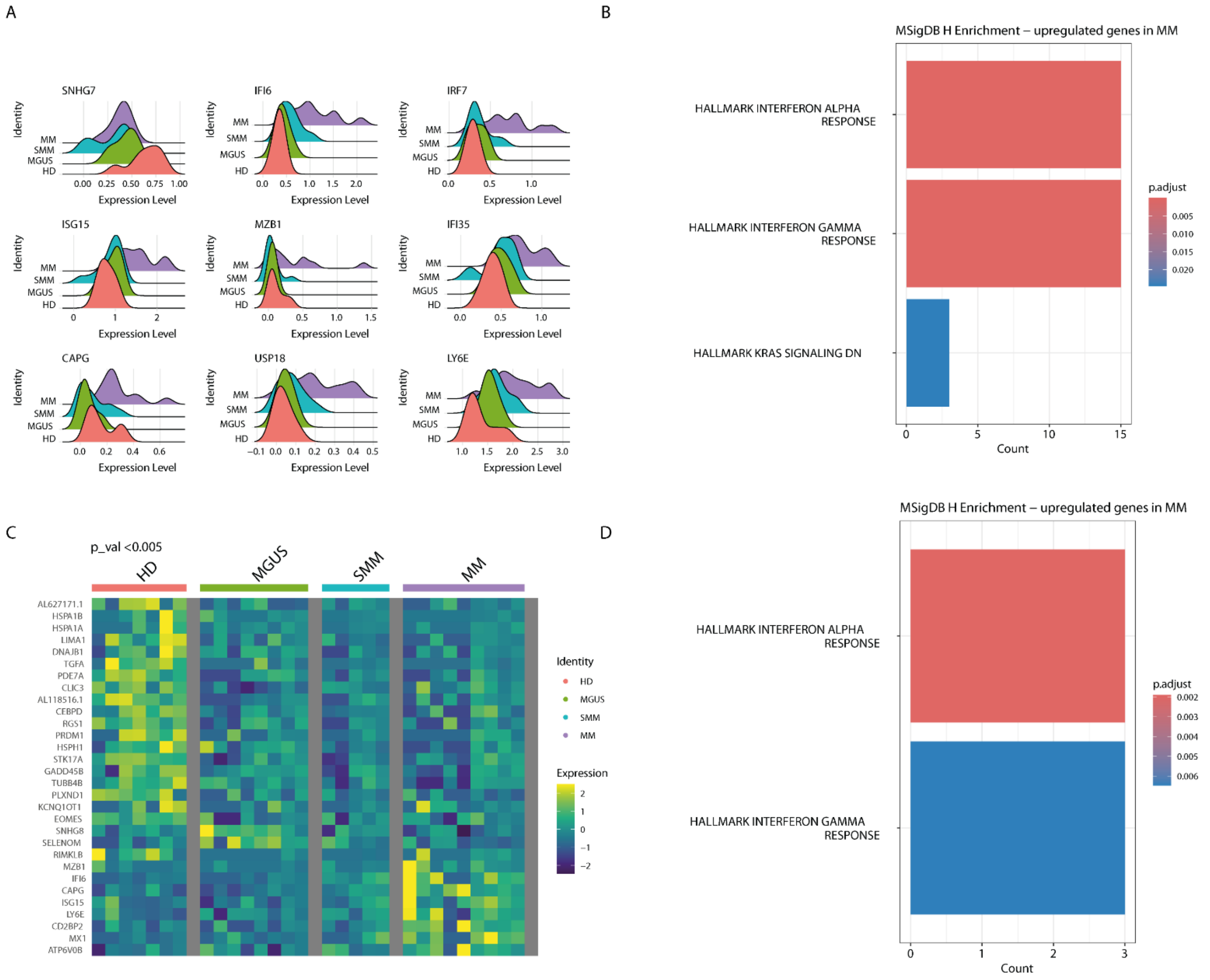

Supplementary Figure S2. ***Enrichment of interferon response pathways in MM.*** (**A**) Density plots of the most significant (adjusted $p < 0.05$) DEGs of total NK (*SNHG7, IFI6, IRF7, ISG15, MZB1, IFI35, CAPG, USP18, LY6E*) between conditions. (**B**) Hallmark pathway enrichment results in total NK for genes upregulated in MM, with bar length indicating gene counts and color representing adjusted *p*-values. (**C**) Heatmap of pseudobulk gene expression for significantly differentially expressed genes ($p < 0.005$) across HD, MGUS, SMM, and MM groups in cluster 1. Columns represent condition-aggregated samples, while rows correspond to the most significant genes. Values are shown as normalized expression levels (color scale from low to high), highlighting distinct expression patterns across disease stages and HD. (**D**) Hallmark pathway enrichment results in cluster 1 for genes upregulated in MM, with bar length indicating gene counts and color representing adjusted *p*-values.

A

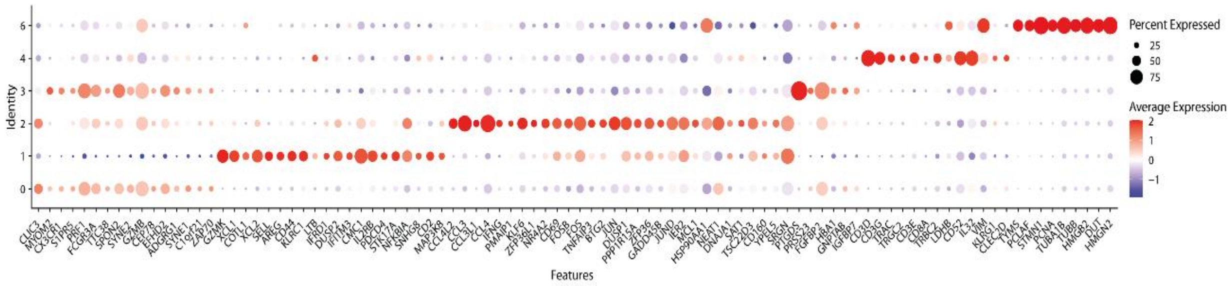

B

Marker Gene Filtering Thresholds

Specific criteria applied to each NK cell cluster

	Filtering criteria			
**NK cluster**	**Adj. p-value**	**avg log2FC**	**pct.1**	**Markers (n)**
0	< 0.05	> 0.5	> 0.20	16
1	< 0.05	> 1	> 0.25	20
2	< 0.05	> 1	> 0.25	15
3	< 0.05	> 0.5	> 0.20	15
4	< 0.05	> 0.75	> 0.20	14
6	< 0.05	> 1.5	> 0.50	33

Note: pct.1 represents the percentage of cells expressing the gene in the cluster.

Supplementary Figure S3. ***Identification and filtering of NK cell cluster marker genes.*** (**A**) Dot plot showing the expression of selected marker genes across NK cell clusters. Each dot represents a gene–cluster pair, with dot size indicating the percentage of cells within the cluster expressing the gene and color representing the average scaled expression level (z-score). This visualization highlights both the specificity and relative expression intensity of cluster-associated markers.(**B**) Summary table of marker gene filtering criteria applied to each NK cell cluster, including adjusted *p*-value, average log2 fold change, and minimum percentage of expressing cells (*pct.1*). The total number of selected marker genes per cluster is reported.

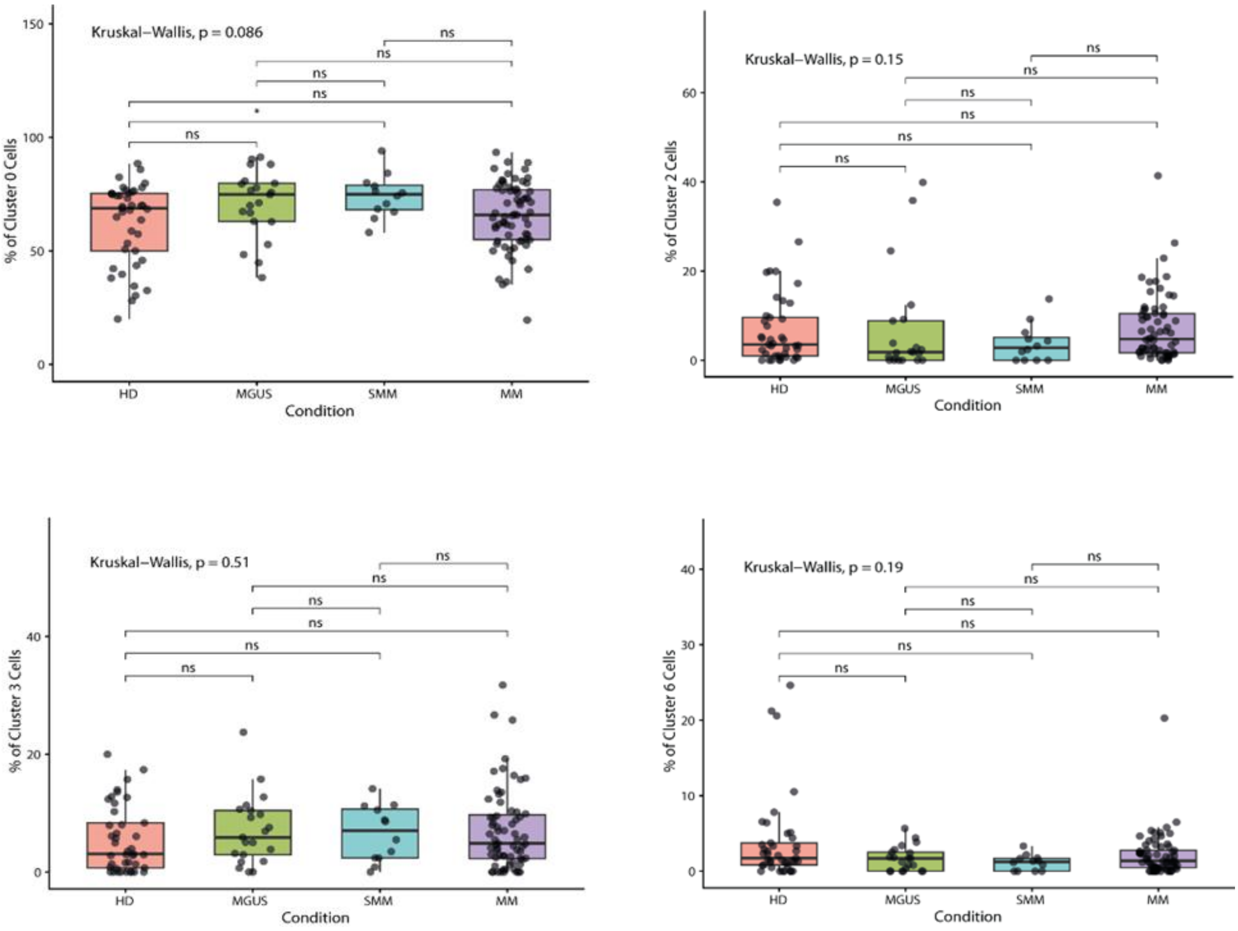

Supplementary Figure S4. ***Relative abundance of NK clusters 0, 2, 3, 6 across disease stages.*** Box plots showing the frequency of individual NK cell clusters expressed as the percentage of each cluster within the total NK compartment at the patient level (minimum of 30 NK cells per patient). Each dot represents an individual patient. Comparisons across HD, MGUS, SMM, and MM were assessed using the Kruskal–Wallis test, and pairwise comparisons were performed using Wilcoxon rank-sum tests (ns: $p > 0.05$; *: $p \leq 0.05$).

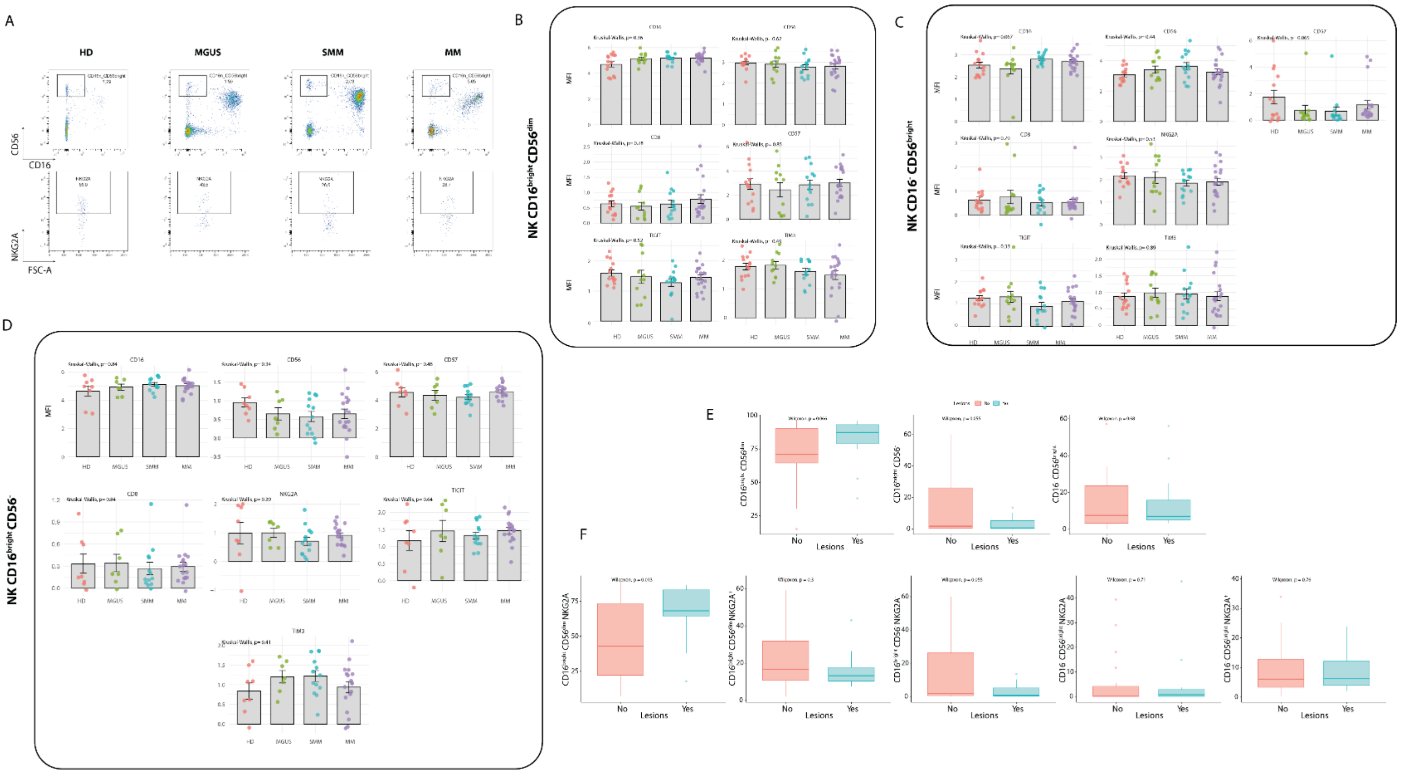

Supplementary Figure S5. ***Flow cytometric characterization of NK cell subsets across disease stages.*** (**A**) Representative flow cytometry dot plots showing the gating strategy and distribution of NK cells across disease stages. (**B**–**D**) Quantification of the main bone marrow NK-cell subsets across clinical groups (HD, MGUS, SMM, and MM), stratified according to CD16 and CD56 expression and further resolved based on the phenotypic markers analyzed. Data are shown as boxplots, with each dot representing an individual sample. In figures (**E**,**F**), boxplots compare NK cell subsets in MM samples according to the presence of bone lesions (No vs Yes), showing the relative abundance of the different populations. Statistically significant differences are indicated in the corresponding panels.

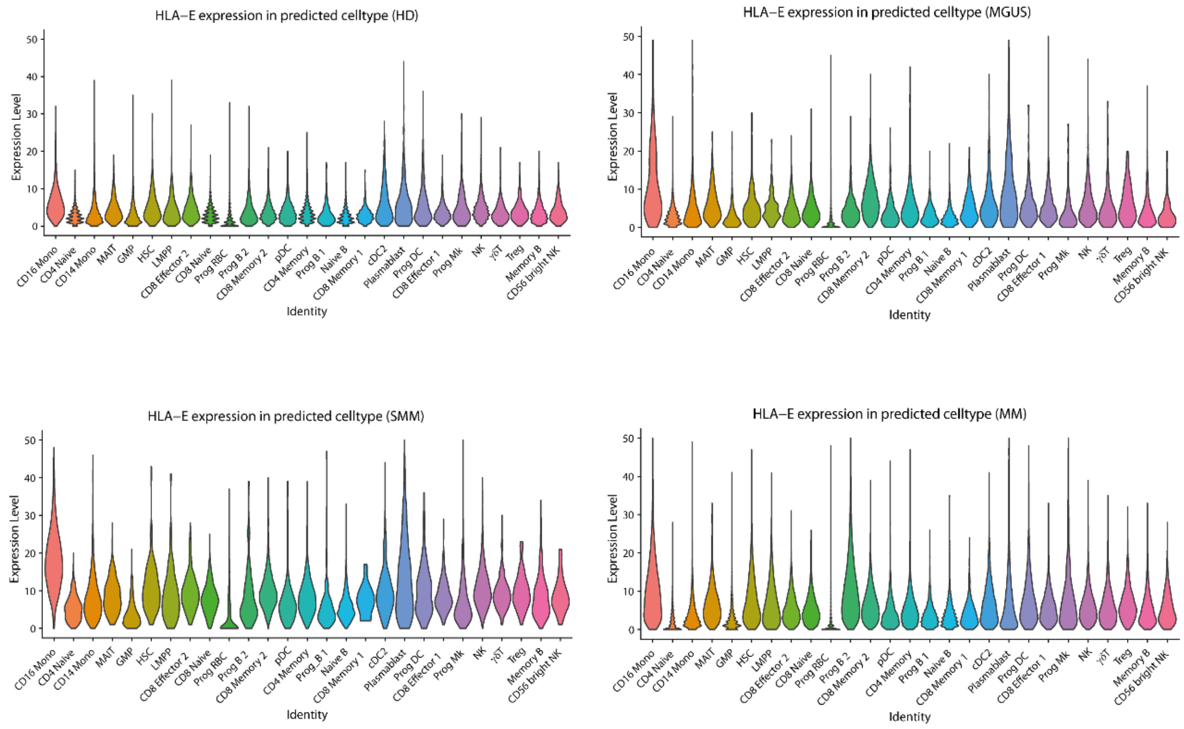

Supplementary Figure S6. ***Cell type–specific HLA-E expression across disease stages.*** Violin plots showing the distribution of HLA-E expression across predicted bone marrow cell types in HD,

MGUS, SMM, and MM. Expression levels are shown for each annotated cell type, highlighting cell type–specific and stage-dependent differences in HLA-E expression.

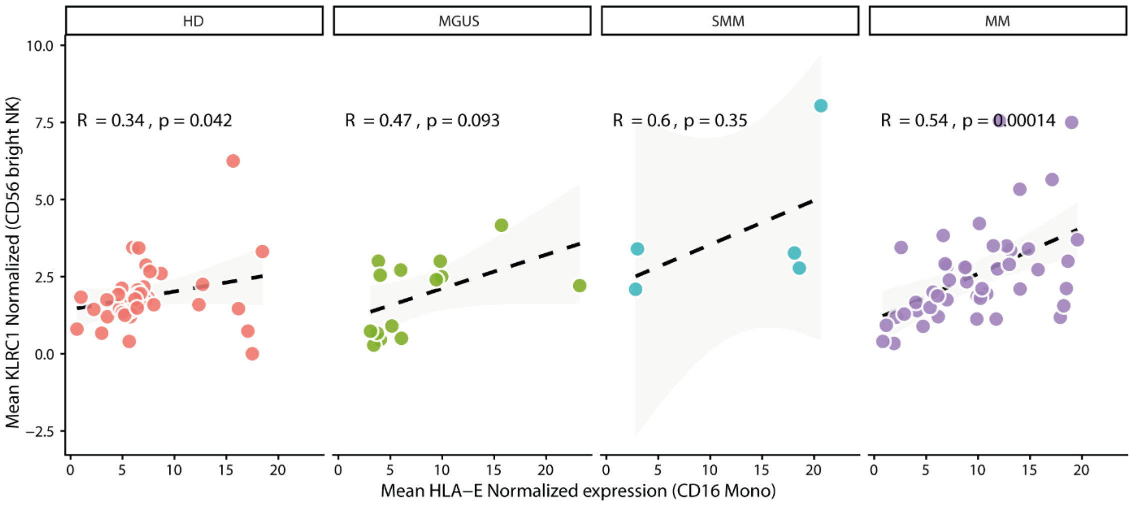

Supplementary Figure S7. ***Stage-dependent modulation of the HLA-E-NKG2A axis.*** The figure shows patient-level correlation analyses between mean HLA-E expression in CD16⁺ monocytes and mean *KLRC1* expression in CD56^{bright} NK cells within each condition; each point represents one patient (minimum of 5 cells per cell type).

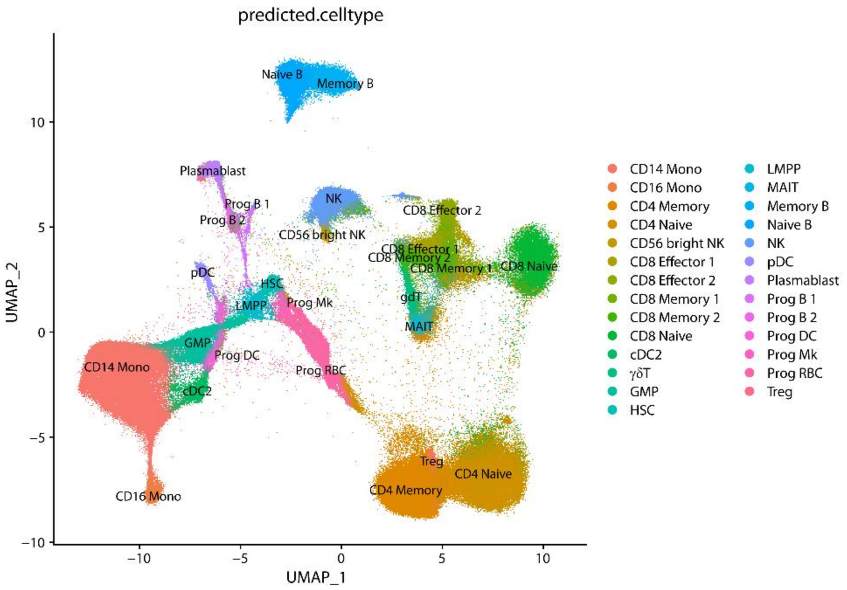

Supplementary Figure S8. ***UMAP representation of predicted bone marrow cell types.*** UMAP visualization of scRNA-seq data showing predicted cell types across the bone marrow compartment. Each point represents a single cell, colored by annotated cell identity based on transcriptional profiles, including myeloid, lymphoid, progenitor, and erythroid lineages. The plot highlights the organization and relative separation of major immune and hematopoietic populations, as well as transitional and progenitor states.

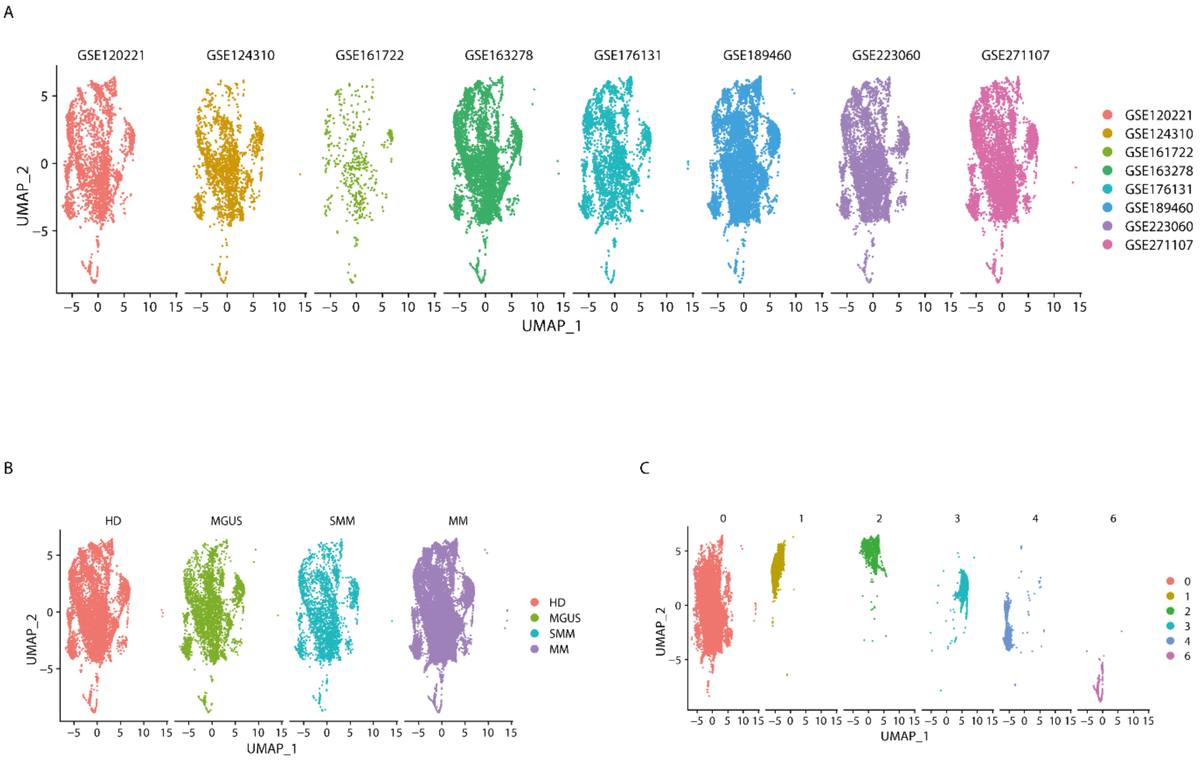

Supplementary Figure S9. ***UMAP integration of datasets, clinical conditions, and RPCA-based clustering.*** (**A**) UMAP embedding of the integrated single-cell data colored by GEO study of origin (GSE120221, GSE124310, GSE161722, GSE163278, GSE176131, GSE189460, GSE223060, GSE271107), illustrating effective batch integration. (**B**) UMAP representation of the same cells colored by clinical condition (HD, MGUS, SMM, MM). (**C**) UMAP colored by RPCA-derived clusters, highlighting transcriptionally distinct cell populations within the integrated dataset.

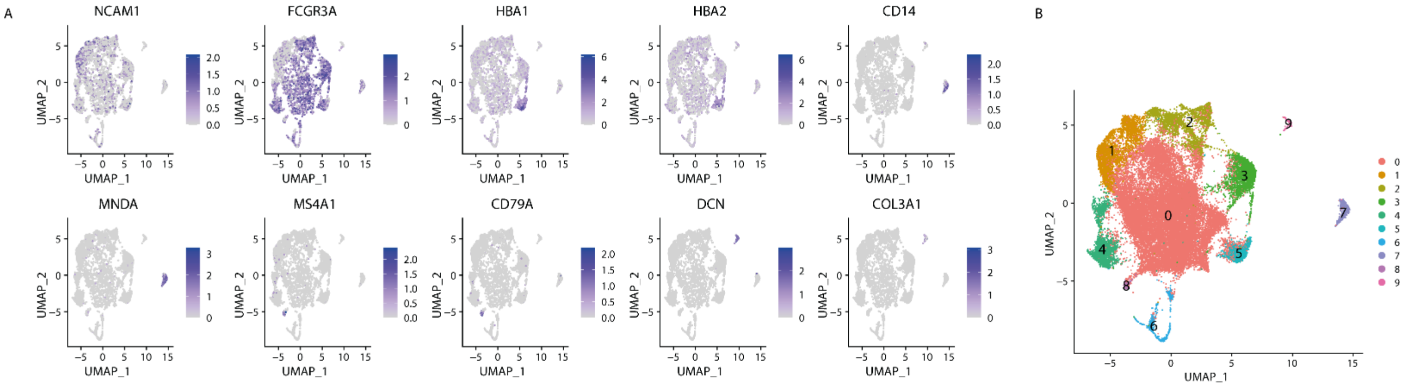

Supplementary Figure S10. ***UMAP-based visualization of cell populations and marker gene expression.*** (**A**) Feature plots showing the expression levels of selected marker genes (*NCAM1, FCGR3A, HBA1, HBA2, CD14, MNDA, MS4A1, CD79A, DCN, COL3A1*) projected onto the UMAP embedding derived from RPCA-reduced data. Color intensity reflects normalized gene expression. (**B**) UMAP embedding colored according to unsupervised cluster assignments, identifying distinct cell populations (clusters 0–9) in the same low-dimensional space.
